# Large Extracellular Vesicles: Have We Found the Holy Grail of Inflammation?

**DOI:** 10.3389/fimmu.2018.02723

**Published:** 2018-12-13

**Authors:** Artur Słomka, Sabine Katharina Urban, Veronika Lukacs-Kornek, Ewa Żekanowska, Miroslaw Kornek

**Affiliations:** ^1^Department of Pathophysiology, Nicolaus Copernicus University in Toruń, Ludwik Rydygier Collegium Medicum, Bydgoszcz, Poland; ^2^Department of Medicine II, Saarland University Medical Center, Saarland University, Homburg, Germany; ^3^Institute of Experimental Immunology, University Hospital of the Rheinische Friedrich-Wilhelms-University, Bonn, Germany; ^4^Department of Oncology, Hematology and Rheumatology, University Hospital Bonn, Bonn, Germany

**Keywords:** microvesicles and exosomes, inflammation, platelet-derived microvesicles, leukocyte-derived microvesicles, endothelial-derived microvesicles

## Abstract

The terms microparticles (MPs) and microvesicles (MVs) refer to large extracellular vesicles (EVs) generated from a broad spectrum of cells upon its activation or death by apoptosis. The unique surface antigens of MPs/MVs allow for the identification of their cellular origin as well as its functional characterization. Two basic aspects of MP/MV functions in physiology and pathological conditions are widely considered. Firstly, it has become evident that large EVs have strong procoagulant properties. Secondly, experimental and clinical studies have shown that MPs/MVs play a crucial role in the pathophysiology of inflammation-associated disorders. A cardinal feature of these disorders is an enhanced generation of platelets-, endothelial-, and leukocyte-derived EVs. Nevertheless, anti-inflammatory effects of miscellaneous EV types have also been described, which provided important new insights into the large EV-inflammation axis. Advances in understanding the biology of MPs/MVs have led to the preparation of this review article aimed at discussing the association between large EVs and inflammation, depending on their cellular origin.

## Extracellular Vesicles at a Glance

The story of EVs started in 1946, when Chargaff and West (1) reported that prolonged centrifugation of human plasma (31,000 × g per 150 min) resulted in extending the coagulation time due to loss of the “clotting factor.” In 1967, Wolf (2) identified platelet-derived vesicles, which were named “platelet dust”—his paper is considered a milestone in EV research by many authors. Fourteen years later, Trams and colleagues (3) for the first time used the term “exosomes” to describe vesicles released from normal and neoplastic cell cultures. A series of studies by Johnstone et al. focusing on the role of exosomes during blood reticulocyte maturation (4–6) also need to be mentioned, in which the authors concluded that exosome shedding leads to loss of some plasma membrane functions (4) due to the elimination of redundant membrane proteins (6). Undoubtedly, the findings of these studies helped to understand that exosomes may perform crucial roles in cellular functioning and that they are not just cellular remainder.

In recent years, there have been major advances in the understanding of the biology of EVs. However, the accurate definition of EVs is still a matter of debate. There are many reasons why it is still difficult to establish a clear, meticulous definition of EVs, for example the fact that they are released from many cell types which results in their varied compositions and functions. Moreover, they are released via multiple mechanisms. Also, EVs exhibit various sizes (30–2,000 nm in diameter), therefore many different analytical methods are used for their isolation and identification from the extracellular milieu (7).

In late 2014, the International Society for Extracellular Vesicles (ISEV) published a statement paper on minimal experimental requirements for the definition of EVs and their functions (8). The authors present basic steps of the research that are required for obtaining accurate results on EVs, including separation, characterization and functional studies (8). However, these recommendations should be continually reviewed (9).

Another aspect that needs to be emphasized is that, starting from 2004, the most common term for EVs used in literature is the “exosome” (or “exosomes”) (8), however many other terms are also applied to describe EV subtypes; the terminology is constantly evolving. Currently, in order to systematize the knowledge on EVs, three main EV types are recognized: exosomes, microvesicles (MVs; microparticles, MPs; ectosomes), and apoptotic bodies (apoptotic vesicles) (10). All of these EV subtypes have common denominators, for example: they are nano-sized vesicles composed of phospholipid bilayers with a spheroidal shape and contain membrane and cytosolic proteins, receptors, and nucleic acids originating from their cell of origin (11). Notwithstanding, the diversity of EV antigens can also be considered a feature that differentiates EVs in terms of cellular origin and functions. Indeed, an Internet compendium of exosomal cargo, ExoCarta, (http://www.exocarta.org) contains data on 41,860 proteins, over 7,540 RNAs and 1,116 lipids identified in exosomes in multiple organisms (12). Two other online databases, EVpedia (http://www.evpedia.info) (13) and Vesiclepedia (14) (http://www.microvesicles.org) present information on all EV types including, but not limited to, exosomes.

Recently, ISEV proposed to classify EVs on the basis of centrifugation conditions into: EVs sedimenting at 100,000 × g into small EVs (sEVs) rather than exosomes; EVs sedimenting at speeds lower than 20,000 × g into medium EVs (mEVs, microvesicles, ectosomes), and EVs sedimenting at 2,000 × g into large EVs (lEVs, large fragments of cells, large apoptotic bodies) (15). Since these recommendations are relatively new (dated March 2017), most authors still use previous, long-established terms. Numerous excellent reviews on EV biogenesis, including their formation and secretion, have already been published (16, 17), thus, in the current review, we provide only a brief description of the two main EV populations: small (exosomes) and medium EVs (microvesicles).

## Exosomes—a Short Presentation

**Figure 1:**
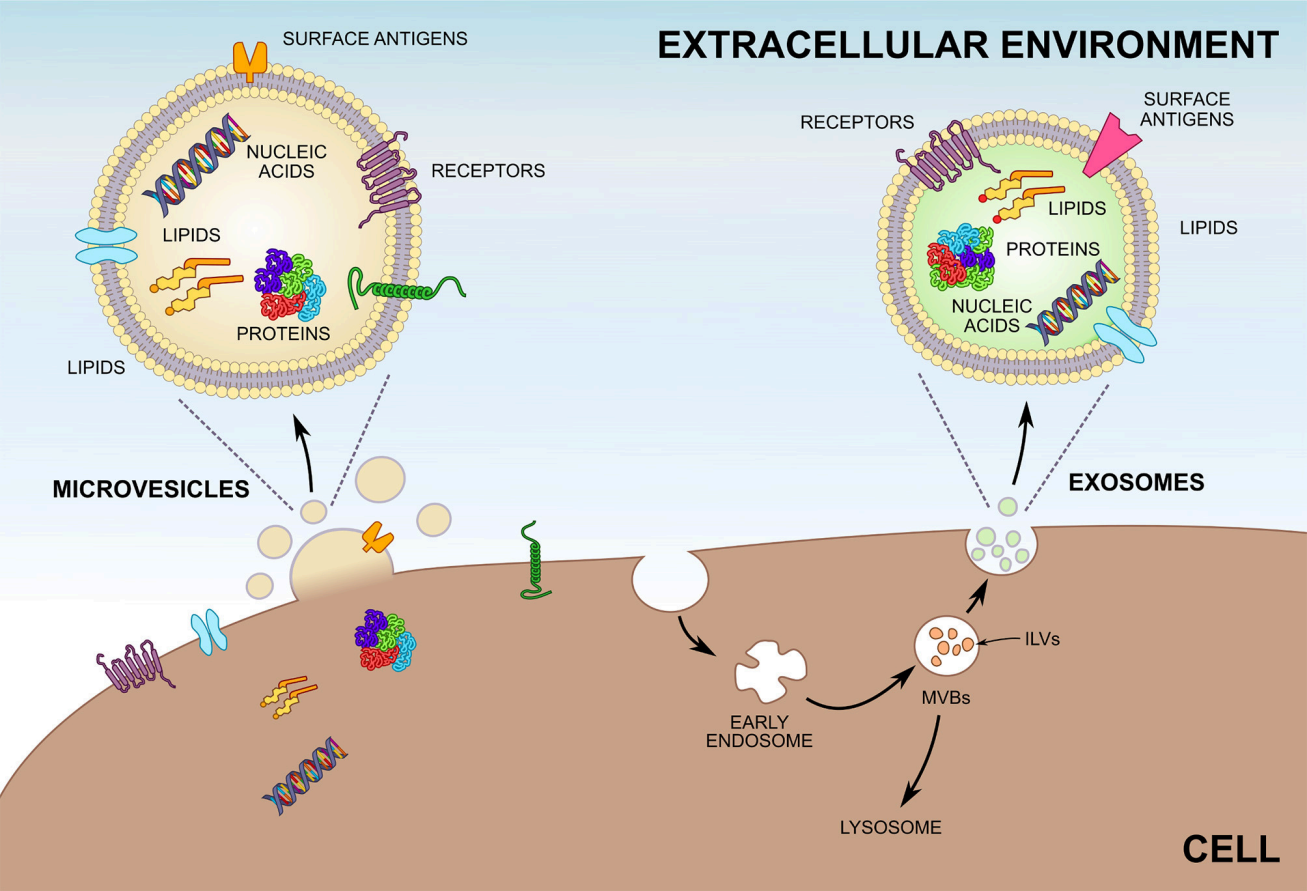
Biogenesis of microvesicles (MVs) and exosomes. Unlike MVs, which are shedded directly from the plasma membrane, most exosomes are formed by invagination of endosomes and are stored within multivesicular bodies (MVBs) before release. Exosomes inside MVBs are also called intraluminal vesicles (ILVs). Upon fusion of MVBs with the plasma membrane exosomes are released into the extracellular environment. Both MVs and exosomes enclose greatly varying compositions of proteins, lipids, and nucleic acids and can be characterized by differing surface antigens.

Exosomes are the smallest among all the EV subtypes (30–150 nm) and their density ranges between 1.10 and 1.19 g/mL. They are secreted by many physiological cell types (18–37) summarized in Table 1. The presence of exosomes in different biological fluids is well researched (38–52); cancer cells are also known to have the ability to release exosomes (53). Most exosomes are secreted from multivesicular bodies (MVBs) (16), also known as multivesicular elements (MVEs), late endosomes or endocytic carrier vesicles (54, 55). *In vivo* experiments elegantly demonstrated that MVBs are organelles containing intraluminal vesicles (ILVs), which release exosomes into the extracellular space upon fusion with the plasma membrane (54). In contrast, T cells may release exosomes directly from discrete domains of the plasma membrane (56). Two sophisticated mechanisms are engaged in exosome generation. One of them depends on the ESCRT (endosomal sorting complex required for transport) machinery (57), while the other one is ESCRT-independent (58). Naturally, not all ILVs become exosomes, since part of MVBs fuse with lysosomes and undergo destruction (Figure 1) (58). Tetraspanins (CD9, CD63, CD37, CD81, CD82), heat shock proteins (HSPs), tumor susceptibility gene 101 protein (Tsg101), and ALG-2-interacting protein X (Alix) are all antigens commonly expressed on the exosomes surface (11, 59). With reference to ExoCarta (12), CD9 is the major exosomal antigen identified in 98 different studies. Importantly, basic studies conducted in the past several years have confirmed that exosomes are predominantly involved in cell-to-cell interactions (60–62).

**Table 1:** Exosome characteristics according to type of parental cell.

**Type of cell releasing exosomes**		**Size (nm)**	**Density (g/mL)**	**Exosome-specific antigens**		**References**
Blood cells	Platelets	40–100	1.14–1.18	a. CD63—classic for many exosomes.		(18)
	B lymphocytes	60–80	1.13	a. major histocompatibility complex class II (MHC class II).		(19)
	Monocytes	50–100	ND	a. miRNA-223.		(20)
	Neutrophils	30–80	ND	a. Asthma remodeling-related proteins, including: Matrix metallopeptidase 9 (MMP9), Leukotriene A4 hydrolase (LTA4H), Serpin family H member 1 (SERPINH1), Collagen type I alpha 1 chain (COL1A1).		(21)
	Eosinophils	162 ± 13.6	ND	a. ALG-2-interacting protein X (Alix), b. CD63, c. CD9.		(22)
Central nervous system cells	Microglia	40–120	1.15	a. Membrane alanyl aminopeptidase (ANPEP), b. Monocarboxylate transporter 1 (MCT-1).		(23)
	Oligodendrocytes	30–80	1.10–1.14	a. Myelin proteolipid protein (PLP), b. 2′3′-cyclic-nucleotide-phosphodiesterase (CNP), c. Myelin basic protein (MBP), d. Myelin oligodendrocyte glycoprotein (MOG).		(24)
	Cortical neurons	100	1.11–1.19	a. Glutamate/aspartate anionic amino acid transporter 1 (GLAST1), b. Ceruloplasmin.		(25)
Dendritic cells		30–100	ND	a. Tumor necrosis factor alpha (TNF-α).		(27)
		30–100	ND	a. MHC class I and class II, b. CD80, CD86, CD40, CD14.		(28)
Adipocytes		50–150	ND	a. Matrix metalloproteinase-3 (MMP3).		(30)
Mast cells		40–80	ND	a. 116 miRNAs, b. 1,800 mRNAs.		(31)
		30–100	ND	a. 82 mast cell-specific proteins, b. Mast cell-specific transcripts, including: c. Mast cell carboxypeptidase A (CPA3), d. Tryptase alpha/beta 1 (TPSAB1).		(32)
Endothelial cells	Human umbilical vein endothelial cells (HUVECs)	30–150	ND	a. Different miRNAs: miR-21, miR-126-3p, miR-126-5p, miR-222.		(33)
	Human brain microvascular endothelial cells (HBMECs)	< 200	ND	a. CD105, b. CD144.		(34)
Endothelial progenitor cells (EPCs)		< 200	ND	a. CD34, b. Kinase insert domain receptor (KDR).		(34)
Hepatocytes		57.6 ± 23 and 49.5 ± 17	ND	a. 251 proteins, including: b. Cytochromes, c. Uridine 5′-diphospho-glucuronosyltransferase (UGT), d. Apolipoprotein E (ApoE).		(35)
Intestinal epithelial cells		30–90	ND	Apical exosomes: a. MHC class I and class II, b. CD26, c. Syntaxin 3 (STX3), d. Microsomal dipeptidase (MDP).	Basolateral exosomes: a. MHC class I and class II, b. CD26, c. A33 antigen, d. Epithelial cell surface antigen (ESA).	(36)
Cardiomyocytes		~100	ND	a. Glucose transporters (Glut1, Glut4), b. Lactate dehydrogenase (LDH), c. Glyceraldehyde-3-phosphate dehydrogenase (GAPDH).		(37)

*ND, not determined*.

## Microvesicles (Microparticles)—a Short Presentation

Each EV type is unique with regard to size and biogenesis. Analysis of MVs from human cells and cell cultures reveal that they are plasma membrane vesicles with diameters ranging from 100 to 1,000 nm (1 μm) (17). Nevertheless, similarly to exosomes, a uniformly accepted definition of MVs is not available. One noteworthy description was proposed by Shet et al. (63), who characterized MVs as vesicles: obtained via ultracentrifugation, with a size of ≤ 1000 nm and expressing phosphatidylserine (PS) verified by annexin V-positive staining. In contrast, the group of Connor et al. (64) described the existence of annexin V-negative MVs. Of note, some authors studying MVs define them as exosomes or use these two concepts as synonyms, which is incorrect. Under both physiological and pathological conditions, MVs are released from cytoplasmic membrane, and engrossingly, the same cells may produce exosomes and MVs concurrently (18, 21). MVs may be released from multiple cell types, including platelets (18), erythrocytes (65), leukocytes [neutrophils (21, 66), monocytes (67), T, and B lymphocytes (67)], brain cells (68, 69), dendritic cells (70), adipocytes (71), endothelial cells (72, 73), endothelial progenitor cells (74), hepatocytes (75), and by hardly ever researched photoreceptors (76) as well as by tumor cells (16, 17). Subsequently, numerous reports describe the occurrence of MVs in biological fluids. Typically, peripheral blood (67) is a standard material for MV isolation and characterization. Other MV-containing fluids are cord blood (77), urine (78), cerebrospinal fluid (79), saliva (80), amniotic fluid (81), synovial fluid (82), and vitreous fluid (76). Despite the naturally occurring biological fluids, evidence suggests that MVs are present in bronchoalveolar lavage fluid (BALF) (83), ascites, pleural, chyloid, and postoperative drainage fluid (84), and likewise can be isolated from atherosclerotic plaques (85). Data gathered from experimental and clinical investigations have implied that MVs are shedding from plasma membranes upon cell activation and apoptosis and that the antigenic expression of endothelial (86), platelet (87), and monocytic (88) MVs depends on the type of stimulus. Given that MVs are fragments of cell membranes, it might seem that their release does not require convoluted biochemical processes. In reality, they are multi-stage processes the mechanisms of which are not yet fully understood (89). As can be deducted from Table 2, antigens of parental cells can be used to identify MVs in biological fluids as well as in conditioned media from cultured tissues. Moreover, the results of *in vitro* and *in vivo* studies, although not unanimously (64), suggest that the vast majority of MVs expose PS. The review of literature also shows that many scientists largely focused their attention on another MV surface antigen, namely tissue factor (TF). Thus, TF-bearing MVs are increasingly being used to evaluate thromboembolic complications in different pathological conditions (90), including cardiovascular diseases (91, 92) and cancer (93). The great variety of bioactive molecules (proteins, lipids, and nucleic acids) which can be transported by MVs from cell to cell enables these nano-sized particles to perform many functions in coagulation, inflammation, cancer, and angiogenesis (94). In this paper we will review current state of knowledge on the role of MVs in inflammation and inflammatory-related disorders.

**Table 2:** Microvesicle characteristics according to type of parental cell.

**Type of cell releasing microvesicles**		**Size (nm)**	**MV-specific antigens**	**References**
Blood cells	Platelets	100–1000	a. Glycoprotein Ib (GPIb, CD42b), b. Glycoprotein IIb/IIIa (GPIIb/IIIa, α_IIb_β_3_, CD41a), c. P-selectin (CD62P), d. Platelet endothelial cell adhesion molecule (PECAM-1, CD31), e. Integrin β_1_ (CD63).	(18)
	Erythrocytes	< 1000	a. Glycophorin A (GYPA, CD235a), b. Glycophorin B (GYPB, CD235b), c. Blood group antigens (RH, KEL, JK, FY, LE, LU).	(65)
	Neutrophils	< 1000	a. Carcinoembryonic antigen-related cell adhesion molecule 8 (CEACAM8, CD66b), b. L-selectin (CD62L), c. Myeloperoxidase (MPO).	(66)
	T lymphocyte	< 1000	a. CD3.	(67)
	B lymphocyte	< 1000	a. CD19.	(67)
	Monocytes	< 1000	a. CD14. b. Tissue factor (TF).	(63)
Central nervous system cells	Glia	300–1000	a. P2Y12, b. CD45.	(68)
		< 1000	a. GFAP, b. Glutamate transporter 1 (GLT-1), c. TF.	(69)
	Neurons	< 1000	a. Neuron-specific enolase (NSE), b. Na^+^/K^+^ ATPase α3, c. TF.	(69)
Dendritic cells		170 (mean)	a. Alpha-actinin 4 (ACTN4).	(70)
Adipocytes		30–500	a. Fatty acid binding protein 4 (FABP4), b. Adiponectin, c. Perilipin A/B.	(71)
Endothelial cells	Human umbilical vein endothelial cells (HUVECs)	100–1500	a. E-selectin (CD62E), b. Intercellular adhesion molecule 1 (ICAM-1, CD54), c. PECAM-1, d. Integrin αvβ3. e. TF, f. Thrombomodulin (TM, CD141).	(72)
	Human brain microvascular endothelial cells (HBMECs)	< 1000	a. Endoglin (CD105), b. ICAM-1, c. VCAM-1, d. MHC class I and II, e. CD40, f. Inducible T-cell costimulator ligand (ICOSL, CD275).	(73)
Endothelial progenitor cells (EPCs)		< 1000	a. ICAM-1, b. Integrin α4, c. Integrin β1 (CD29), d. CD44.	(74)
Hepatocytes		100–1000	a. Maltase-glucoamylase (MGA), b. Ceruloplasmin precursor, c. Amine oxidase, copper containing 3 (AOC3), d. Apolipoprotein E precursor, e. Vitamin D-binding protein precursor, f. Isocitrate dehydrogenase 1, soluble (IDH1), g. Fumarylacetoacetate hydrolase (FAH), h. Vanin-1 (VNN1), i. Transforming growth factor, beta-induced (TGFBI).	(75)

## Platelet-derived Microvesicles (PMVs) and Inflammation

Platelets are small, anucleated cellular elements derived from megakaryocytes which play substantial role in blood coagulation (95). It is generally recognized that platelets are effectively the root cause of circulating MVs. As discussed by Kornek and Schuppan (96), platelet-derived MVs (PMVs) constitute the most commonly researched MV type. Furthermore, PMVs represent predominant fraction of MVs in circulation (97). Flow cytometry is commonly used to determine the number of PMVs in biological fluids by using monoclonal antibodies against glycoprotein IIb (CD41), glycoprotein IIIa (CD61), and P-selectin (CD62P) (98).

This section of the article contains the summary of current state of knowledge on the role of PMVs in inflammation, although they can also be found in biological fluids of healthy organisms. In order to improve understanding of PMVs' role in physiological conditions, Berckmans et al. (97) conducted a study which showed that PMVs have anticoagulant properties thanks to protein C (PC) activation. It is also interesting that the function of megakaryocytes as the main source of PMVs in healthy subjects was confirmed in another research (99).

Two types of observations point at the significance of PMVs in inflammation. First, a number of experimental researchers reported that PMV cargo can interact with cells involved in inflammatory reactions. Second, an increase in blood PMV levels has been reported in inflammatory-associated disorders. Essentially, the proinflammatory action of PMVs comes down to modulation of several processes, including activation of both immune cells and endothelium, intensification of leukocyte transendothelial migration (TEM) and cell to cell interaction, stimulation of chemotaxis, and reducing apoptosis of inflammatory cells. Moreover, PMVs remain a rich potential source of proinflammatory cytokines and complement components. At the beginning of the MV era it was comprehensively accepted that PMVs are strong proinflammatory mediators. This is partially true and also obviously incomplete since recent studies documented that PMVs serve as anti-inflammatory factors (11, 16, 94, 96). This missing aspect will be discussed in the subsequent part of this section.

One universal concept that has emerged from previous studies is that PMVs modulate the phenotype of different cells via transport of their bioactive components to target cells. The observation that PMVs can activate a great variety of cells which are engaged in both immunity and inflammation allowed major progress in the understanding of interdependence between inflammatory processes and PMVs. This mechanism was studied in detail by Barry et al. (100, 101) in the late 90s. In the initial report, they proved that PMVs induce expression of cyclooxygenase-2 (COX-2) and prostacyclin (PGI_2_) production in endothelial cells (100) throughout arachidonic acid (AA). This observation initiated a series of studies on the possibility of shifting endothelial properties into a proinflammatory state with PMV participation. The authors further noted that interaction between monocytes and endothelial cells is modulated by PMVs (101). This data clearly indicates that PMVs have the ability to activate intracellular cell adhesion molecule-1 (ICAM-1, CD54) on endothelial cells and integrin subunit alpha L (CD11a), integrin subunit alpha M (CD11b), and CD14 on blood monocytes as well as on the U-937 macrophage cell line (101). All of these surface antigens are crucial in inflammation. ICAM-1 interacts with two types of leukocyte receptors: lymphocyte function-associated antigen 1 (LFA-1, CD11a-CD18) and macrophage-1 antigen (Mac-1, CD11b-CD18), which is the key step leading to TEM (102). This complex process, so called diapedesis, recruits leukocytes to the site of inflammation (103). Interestingly, the chemotaxis of U-937 cells also seems to be induced by PMVs (101). When examining the mechanism of monocyte recruitment to endothelium, Mause et a1. (104) reported that this process depends on chemokine (C-C motif) ligand 5 [CCL5, also known as regulated on activation, normal T cell expressed and secreted (RANTES)], transferred into endothelial cells by PMVs. Accordingly, an early study was conducted in order to understand the relationship between PMVs and leukocyte-leukocyte interactions (105). Forlow et al. (105) demonstrated that P-selectin, which is a protein localized in the membranes of PMVs, constitutes a critical component in neutrophil aggregation and accumulation. After creating a specific bond with its primary ligand, P-selectin glycoprotein ligand-1 (PSGL-1, CD162) it serves as “bridge” between circulating or adherent neutrophils (105). Other researchers proved that PMVs inhibit the apoptosis of polymorphonuclear leukocytes (PMNs) (106). Indeed, stimulation of platelets with thrombin leads to the release of PMVs, which through transforming growth factor beta 1 (TGF-β1) suppress PMNs apoptosis (106). On the other hand, PMVs promote apoptosis in macrophages, probably because of the transfer of active caspase 3 (107).

The PMVs' ability to induce the adhesion of PMNs to endothelium spurred considerable interest, mostly because this process is involved in inflammatory reactions (as mentioned earlier). A study conducted by Lindemann et al. (108) concluded that interleukin (IL)-1β is carried by PMVs and induces human endothelial cell adhesiveness for neutrophils. Recently, Xie et al. (109) attempted to characterize the role of PMVs in endothelial cell damage. Data published by them have shown that PMVs can synergize with PMNs and together are responsible for the destruction of microvascular endothelium (109). Most notably, this reaction is dynamically modulated by the interaction of soluble CD40 ligand (sCD40L) from PMVs with CD40, a receptor found on granulocytes (109). Recognition of PMVs as a source of sCD40L is extraordinarily important for considering their role in inflammation. Despite the findings of Xie et al. (109), sCD40L is a strong proinflammatory molecule, which binds to CD40 in cells of the immune system, such as monocytes/macrophages, as well as in endothelial cells, and initiates a series of biochemical and molecular reactions, including: monocyte extravasation, cytokine synthesis [monocyte chemoattractant protein-1 (MCP-1), IL-1, IL-6, IL-8, matrix metalloproteinases (MMPs)], and reactive oxygen species (ROS) generation (110). Furthermore, proteomic analysis of PMVs revealed that they constitute a source of many proinflammatory compounds (111). While literally hundreds of proteins were detected (111), those engaged in inflammatory response belonged to the C-X-C motif chemokine family (CXCL4, CXCL7) and the C-C motif chemokine family (CCL5, CCL23). Furthermore, PMVs were shown to create macromolecular structures with immune complexes (112). Meticulous analysis proved that these structures, via presentation of autoantigens and stimulation of leukotriene production by neutrophils, bolster inflammation (112). It is noteworthy that works of other research groups confirmed that PMVs are a source of IL-1, IL-6 and tumor necrosis factor-α (TNF-α) (113, 114). Without a doubt, the above-described properties of PMVs confirm their active participation in the pathogenesis of atherosclerosis and its complications. Although the formation of atherosclerotic plaques is a complex mechanism, leukocytes activation, adhesion, and migration as well as endothelial dysfunctions are also significant, and now it is clear that all these processes are moderated by PMVs. Accordingly, as might be expected, elevated plasma levels of PMVs were described in atherosclerosis and associated cardiovascular disease (115, 116).

As shown above, much effort has been made to describe the leukocyte-PMV-endothelial axis. Naturally, immunological response is not just a single-step process, but it involves an adaptive immune compartment. Indeed, this question in the context of lymphocyte relationship with PMVs remains substantially underresearched. Notwithstanding, an introductory study demonstrated that PMVs transfer CD40L (CD154) to B cells. CD40L-bearing PMVs induce IgG production, germinal center formation as well as B cell proliferation. Using an immortalized pancreatic endothelial cell line (MS-1), the authors additionally proved that PMVs are actively involved in the regulation of MCP-1 expression (117). More recently, the immunostimulatory effect of PMVs on the acquired immune system was quantified in Daudi B cell line (118). This small study illustrated that PMVs strongly induce the expression of CD86 and CD27 with simultaneous decrease of IgD expression in Daudi cells (118). The synthesis of IgG was increased when Daudi cells were co-cultured with PMVs (118). Although this data is fragmented and remains to be further investigated, the activation of the adaptive immune system is almost certainly related to the cooperation with PMVs.

In recent experiments different agonists, such as thrombin, collagen, and calcium ionophore A23187 (calcimycin) (100, 105), were used to activate platelets and MV release. Notwithstanding, it is well known that platelets may be activated during infection by contact with bacterial proteins. For example, direct evidence implicates that staphylococcal superantigen-like protein 5 (SSL5) is a powerful modulator of PMV generation (119). Major SSL5-induced PMV effects observed were linked with monocytes and provoking them to synthesize proinflammatory cytokines, including IL-1β, TNF-α, MCP-1, and MMP-9 (119). This experiment also showed that PMVs enhance the chemotaxis of monocytes (119). Moreover, PMVs contribute to the development of inflammation during enterohemorrhagic *Escherichia coli*-associated hemolytic uremic syndrome (EHEC-HUS) (120). In the acute phase of the disease patients exhibited increased levels of PMV-expressed complement component 3 (C3) and C9 (120). Release of PMVs rich in complement components and complement control proteins were stimulated by Shiga toxin and lipopolysaccharide (LPS) (120). There is also evidence that PMVs may play a role in viral infections. A study carried out at the University of Louisville provided clear evidence of transferring C-X-C chemokine receptor type 4 (CXCR4) by PMVs to CD4^+^/CXCR4^−^null cells and, in consequence, making them susceptible to infection by human immunodeficiency virus (X4-HIV) (121). It does not come as surprise that Corrales-Medina et al. (122) reported increased levels of PMVs in blood of HIV-infected patients. Furthermore, increased activity of PMVs during HIV infection has been described (123). However, the intensification of PMV generation is not characteristic for all viral infections. For example, patients with dengue virus (DENV) infection exhibit reduced shedding of PMVs (124). The role of PMVs in parasitosis is poorly characterized because researches are mainly limited to malaria infection. The direct engagement of PMVs in *Plasmodium falciparum* infestation was documented by Faille et al. (125). Intriguingly, they have shown that PMVs preferentially bind with *P. falciparum*-parasitized red blood cells (PRBCs) (125). Despite the fact that PMVs can directly bind to human brain endothelial cells (HBECs), PRBCs adherence to HBECs is dramatically increased by PMVs, which links PMVs to cerebral malaria (125). Few clinical studies confirmed this *in vitro* report on the relationship between malaria, its complications and PMVs (126, 127). In particular, high plasma PMV levels were associated with coma depth and thrombocytopenia in patients with *P. falciparum* cerebral malaria (126). Similarly, researchers reported the existence of links between increased levels of plasma PMVs, fever and days with acute illness in *P. vivax* malaria (127). Collectively, these studies consistently demonstrated that acute phase response during infections may be additionally modulated by PMVs.

Traditionally, when thinking about the assessment of inflammation in everyday medical practice, each clinician pays particular attention to C-reactive protein (CRP). CRP is an acute phase reactant with two conformationally different forms: pentameric CRP (pCRP) and monomeric CRP (mCRP), which is the product of pCRP dissociation (128). Even though mCRP is characterized by a stronger proinflammatory potential compared to pCRP (129), previous report demonstrated that out of all the MV types PMVs are the ones to bind pCRP (130). The properties of PMVs escalate localized inflammation through the classical complement pathway activation and leukocyte recruitment into tissues (130).

The mechanism of PMVs role in inflammation is not as simple as it initially appears. Therefore, further insights into the role of PMVs in inflammation originated from previous studies showing their anti-inflammatory attributes (131–135). Unlike well-established proinflammatory effects, the mechanisms of inflammation suppression are poorly understood and, as far as we know, a limited number of papers address this issue. It should also be emphasized that anti-inflammatory properties of PMVs are primarily due to the inhibition of cytokine release. First, PMV cargo serves as signaling molecules to inhibit inflammatory reaction. By way of illustration, lipoxygenase 12 (12-LO) positive PMVs are thought to be involved as mediators in the synthesis of lipoxin A4 (LXA4) by mast cells, which leads to the inhibition of inflammation (131). Second, further insights into the role of PMVs in inflammatory response regulation were gained by demonstration that PMVs reduce the release of the proinflammatory proteins TNF-α and IL-10 by macrophages (132). Simultaneously, the release of TGF-β was induced by PMVs (132). Accordingly, it was speculated that differentiation between monocytes and immature dendritic cells (DCs) is downmodulated by PMVs (132). However, these results should be approached with caution, as PMVs were isolated from stored (not fresh) platelets (132). On the other hand, the ability of PMVs to participate in reprograming macrophage function was also described by Laffont et al. (133). Release of CCL4, TNF, and colony stimulating factor 1 (CSF1) was found to decrease in macrophages co-incubated with PMVs (133). Another investigation confirmed that PMVs reduce TNF-α and IL-8 secretion from plasmacytoid dendritic cells (pDCs) (134). Finally, Dinkla et al. described the inhibitory effect of PMVs on adaptive immune system (135). This study constitutes compelling evidence that regulatory T cells (Tregs) are suppressed to release IL-17 and interferon gamma (IFN-γ) by PMVs in a P-selectin-dependent manner (135). As can be concluded from the analysis of available literature, proinflammatory properties of PMVs constitute the greater part of the paper, whereas anti-inflammatory properties are only analyzed in a scarce number of experimental studies. Nevertheless, PMVs can combine their proinflammatory action with the ability to reduce inflammation.

## Endothelial-derived Microvesicles (EMVs) and Inflammation

It is widely acknowledged that endothelium is a single large organ with weight of around 720 g and surface of 6,000 m^2^ (136). Endothelial cells are an important source of MVs. Altered vascular homeostasis, that is state of activation or apoptosis, is associated with the release of MVs. They are defined based on cytometric analysis of glycoprotein expression, including E-selectin (CD62E), endoglin (CD105), platelet endothelial cell adhesion molecule 1 (PECAM-1, CD31), vascular cell adhesion molecule 1 (VCAM-1, CD106), vascular endothelial cadherin (VE-cadherin, CD144), and melanoma cell adhesion molecule (MCAM, CD146) (137). Some of these surface markers are also characteristic for other MV types (137), therefore their combinations are frequently used in EMV studies, for example CD105^+^/CD144^+^ (138) or CD105^+^/CD146^+^ (139). It was Combes et al. who for the first time provided direct evidence demonstrating the presence of EMVs in circulation (72). Since then many studies were devoted to understanding the role of EMVs in various pathological processes. Considering the origin of EMVs, literature strongly emphasizes their proatherogenic and prothrombotic action; meanwhile, the following section of this review describes the involvement of EMVs in inflammation.

Attention should be drawn to the existence of a disproportion between the number of studies on the role of EMVs in inflammation and the number of reports on PMVs. However, a growing body of evidence indicates that EMVs are also important mediators of inflammatory reactions. Discussing this issue should be initiated by the presentation of *in vitro* results showing that human endothelial cells release MVs after stimulation or injury with various proinflammatory cytokines. An early research conducted by a group of French scientists reported that TNF promotes EMV release by human umbilical vein endothelial cells (HUVECs) (72). Other experiments demonstrated that EMV production is increased in the presence of IL-1α (140), IL-1β (141), IFN-γ (141), complement proteins C5b-9 (142), CRP (143), and LPS (141). The fact that TNF strongly stimulates the release of EMVs is often used in *in vitro* studies (72, 86, 144–147).

The second important remark is that specific set of EMV surface proteins may modulate local and generalized inflammation. It also seems that the expression of these antigens depends on endothelial cell stimulation through inflammatory agents, first and foremost by TNF. Experimental data signify that the TNF-EMV axis is a perfect example of the inflammation cycle: TNF promotes EMV generation, which in turn increases the expression of adhesion molecules on subsequent endothelial cells. In a previously mentioned experiment designed to answer questions about the role of inflammatory stimuli on EMV generation, Combes et al. (72) found that adhesion molecule (E-selectin, ICAM-1, PECAM-1, and αvβ3) expression on EMVs is enhanced by TNF. Moreover, there are indications that TNF-α induction of EMVs depends on p38 mitogen-activated protein kinase (MAPK) (145). Released EMVs act on subsequent endothelial cells, increasing the secretion of soluble ICAM-1, which does not depend on MAPK (145). It should also be stressed that this study associated EMVs with IL-6, demonstrating a very strong positive correlation between these two inflammatory components (145). The findings of Lee et al. (146) confirmed that EMVs increase endothelial surface expression of ICAM-1 in a dose-dependent manner. Among several molecular mechanisms that may contribute to EMV shedding, the authors incontrovertibly proved the role of nuclear factor kappa-light-chain-enhancer of activated B cells (NF-κB) and tumor necrosis factor receptor-1 (TNFR-1) (146). As for the question of inflammation, they also described that attachment of monocytes to endothelial cells is modulated by EMVs (146). Accordingly, an earlier study (147) found the ability of EMVs to bind with monocytes. Indeed, several clinical studies identified increased binding of EMVs with leukocytes in inflammatory conditions, including severe systemic inflammatory response syndrome (SIRS) (148), metabolic syndrome (149), and multiple sclerosis (150, 151). As mentioned earlier, TNF causes increased expression of adhesion molecules, predominantly ICAM-1, both on EMVs and endothelium surface, which results in an intensified interaction between leukocytes and endothelial cells. Moreover, studies show greater affinity of EMVs to monocytes than to neutrophils and lymphocytes (150). It is also clear from experimentally induced TEM that monocyte migration is enhanced when cells are conjugated with EMVs (150, 151). As emphasized at the beginning of this paragraph, interactions between EMVs and endothelial cells are examples of an inflammation cycle. In the same context, Liu et al. (152) have recently confirmed that the axis of TNF-endothelium-EMV-endothelium is a self-perpetuating inflammatory process. They concluded that TNF-induced EMVs stimulate endothelial cells to produce proinflammatory cytokines including interferon gamma-induced protein 10 (IP-10) (152). Interestingly, EMVs can also be generated in the process of endothelial cell stimulation by bubbles, which represents a laboratory model of decompression sickness (DCS) (153). These bubble-induced EMVs support inflammatory responses by promotion of proinflammatory cytokine release (soluble ICAM-1 and soluble VCAM-1) (153). Collectively, the studies discussed in this section lead to the conclusion that EMVs have the ability to activate both endothelium and leukocytes, which fortifies migration of leukocytes to the site of inflammation. Recently, Nakaoka et al. (154) have put forward important and thought-provoking observations on the proinflammatory mechanism of EMV. First, two unique microRNAs (hsa-miR-145-5p and hsa-miR-320a) were encapsulated in EMVs. Second, hsa-miR-145-5p and hsa-miR-320a were transferred to monocytes and upregulated mRNAs of inflammatory cytokines (TNF-α, IL-1β, IL-6, IL-10, and IL-18) (154). In another study by Yamamoto et al. (141) the researchers tried to identify the effect of inflammation-induced EMVs on pericytes. These findings indicate that the release of EMVs in response to inflammatory factors is incorporated into cerebrovascular pericytes and increase vascular endothelial growth factor B (VEGF-B) mRNA and protein expression in a miRNA-dependent manner (141). Unquestionably, this aspect also links EMVs to the process of angiogenesis. On the contrary, experiments reported by Jansen et al. (155) showed that EMVs reduce ICAM-1 expression by transporting miR-222 to endothelial cells. This apparently results in a decrease of monocyte adhesion to endothelium (155). Detailed studies on the mechanisms by which microRNAs associated with EMVs modulate inflammatory reactions are in a relatively early phase of development, however it may already be speculated that the trend in proinflammatory vs. anti-inflammatory properties depends on the type of microRNA.

Only few reports are currently available on the role of EMVs in adaptive immunity modulation. It is important to remember that EMVs activate lymphocyte proinflammatory pathways through surface antigens. Notably, previous work demonstrated that EMVs induce maturation of plasmacytoid dendritic cells (pDCs) (156). PDCs produce great amounts of type I interferons (IFN-α, IFN-β, IFN-ω), type III interferons (IFN-λ1, IFN-λ2, IFN-λ3), as well as IL-6, and TNF-α (157, 158). Thus, it has been convincingly shown that after stimulation with EMVs pDCs secrete IL-6 and IL-8. Fundamentally, this study also identified increased naïve CD4+ T cell proliferation and Th1 cytokine secretion in the presence of EMV-induced mature pDCs (156). Moreover, EMVs support proliferation of CD4+ and CD8+ T cells (73). The presence of molecules engaged in antigen presentation and T cell stimulation, including CD40, major histocompatibility complex (MHC) class I and class II, and inducible T cell costimulator ligand (ICOSL) was reported on EMV surface (73). This finding, combined with the observation that EMVs bind with CD4+ and CD8+ T cells, may provide another explanation on how EMVs modulate immune response. Other observations support the view that EMVs act as activators of T cells response (159, 160). The number of Th1 cells increased when peripheral blood mononuclear cells were co-incubated with EMVs (159). An increase in T-box transcription factor (T-bet) mRNA and protein was documented simultaneously during the same experiment (159). Thus, it may be speculated that EMVs use T-bet to promote Th1 cell differentiation and cytokine synthesis. Subsequent research demonstrated that EMVs deliver miR-155 to T cells (160). Although encapsulated miR-155 does not influence proliferation and apoptosis of T cells, miR-155 inhibition causes suppression of IFN-γ, IL-2, IL-9, and IL-17A release, while increasing the release of other cytokines such as IL-4, IL-6, and IL-10 (160). Clinical implication derived from the above mentioned papers (159, 160) is that changes in T cell functioning under the influence of EMVs constitute critical element of pathophysiology of some disorders such as acute coronary syndrome (ACS) (159) and acute graft-versus-host disease (aGvHD) (160). Accordingly, EMVs may constitute therapeutic target for anti-inflammatory drugs.

Previous investigations contributed to better understanding of dependence between EMVs and the already mentioned well-known acute-phase reactant, CRP. In order to investigate possible interaction between CRP and EMVs, Wang et al. (143) carried out an experiment showing that endothelial cells treated with CRP release more EMVs. This observation is concurrent with data obtained by Devaraj et al. (161). The question arouse if EMVs can carry CRP and what potential biological effect of this phenomenon would be. Experimental and clinical data support potential relationship between EMVs and CRP. Major advance in our understanding of this link came when Habersberger et al. (162) reported that EMVs are involved in the conversion of pCRP to mCRP and transport mCRP to endothelial cells causing their activation as determined by an increase in VCAM-1 surface expression. Crawford et al. (163) recently confirmed that EMVs bear strong proinflammatory mCRP which may enhance TEM of monocytes. Moreover, the number of circulating EMVs correlates with CRP levels in some pathological conditions, such as coronary heart disease (164), chronic kidney disease (165), and familial Mediterranean fever (166).

In the view of pleiotropic nature of EMVs actions, it is not surprising that several studies investigated their roles in infectiology, including bacterial sepsis, viral and malaria infections. Nevertheless, more research needs to be performed in order to gain better understanding of the role of EMVs in bacterial sepsis. Sepsis is a severe and generalized inflammatory reaction in response to infection, which in consequence may lead to increased generation of main types of MVs (167). Another factor critically important for the role of EMVs in sepsis is their relationship with septic complications, primarily with disseminated intravascular coagulopathy (DIC). In a study conducted on adult patients with septic shock due to bacterial infections, Delabranche et al. (168) reported that EMVs may constitute a biomarker for DIC. Moreover, Matsumoto et al. (169) concluded that enhanced generation of EMVs signalizes extensive endothelial injury in sepsis-induced DIC. In contrast, it has recently been shown that MVs isolated from patients with septic shock, including that of endothelial origin, exhibit vasculoprotective effects working against vascular hyporeactivity (170). This may partially explain previous observations made by the group lead by Soriano (171), which described three fundamental pathophysiologic changes associated with sepsis related to EMVs. First, the number of EMVs is increased in sepsis patients in comparison to healthy control group. Second, both EMV and EMV-monocyte conjugate levels are higher in survivors compared with non-survivors. Third, lower levels of EMVs and EMV-monocyte conjugates are associated with organ dysfunction (171). Therefore, an important conclusion of these studies (170, 171) is that EMVs in sepsis may perform a protective role.

Although partial progress has been made in the understanding of the role of EMVs in bacterial infections, little is known about the relationship between EMVs and viral infections. In short, promoting a release of EMVs may be considered common feature of viral infections. For example, elevated numbers of EMVs have been described in HIV-positive patients (172). Othman et al. evaluating the effects of adenovirus administration in mice (173), also reported its ability to generate EMVs. As for other type of human virus, parvovirus B19 (primate erythroparvovirus 1), release of apoptotic EMVs but not activated EMVs have been reported (174). Despite more studies being needed, these reports emphasize the association between endothelial dysfunction and viral infection. Whereas, it is accepted that EMV shedding is linked to bacterial and viral infestation, the mode of EMV action in malaria is not fully understood. One indication that EMVs are involved in malaria pathogenesis comes from a study on children with acute phase of cerebral malaria, showing increased number of EMVs (175). In order to identify the influence of *P. falciparum* infection on EMVs, Wassmer et al. (176) designed a study in which they compared EMV generation in cultures of endothelial cells in patients with uncomplicated and cerebral malaria. After stimulation with TNF, cells obtained from patients suffering from cerebral malaria released significantly more EMVs than cells from uncomplicated cases. Therefore, it is important to find out whether EMVs may be a novel therapeutic target in severe malaria. Studies researching EMVs in infectious diseases provide unique information concerning their role in immunity. Clinically, EMVs may be an excellent biomarker of endothelial dysfunction in various infections. From pathophysiological point of view they perform opposing functions, since they are able to support normal functions of endothelium in sepsis but they are also significant for genesis and evolution of infections complications.

## Leukocyte-derived Microvesicles (LMVs) and Inflammation

Leukocytes (white blood cells, WBCs) gained a considerable interest as a subject of studies by a Nobel Prize winner, Paul Ehrlich (177). It is commonly assumed that they perform essential functions in immunological responses to infections (178). Currently, considerable effort is made to understand how leukocyte-derived microvesicles (LMVs) contribute to hemostasis, inflammation and angiogenesis (179), however this section of the review is intentionally limited to describing the role of LMVs in inflammatory processes.

There is some experimental evidence that LMVs may originate from monocytes (63), neutrophils (66), as well as B and T cells (67). Since this section deals in more detail with the analysis of how LMVs control inflammation, for the sake of convenience it is divided into three paragraphs, one per each cellular source. LMVs' levels are assessed in biological fluids by flow cytometry using specific antibodies against surface proteins (Table 2). Based on literature review, we claim that LMVs can act as either pro- or anti-inflammatory modules. While the proinflammatory effects are relatively well understood, the contrary effects are much less described.

Early investigations of possible roles of LMVs in inflammation concluded that leukocytes release MVs in response to stimulation by chemotactic peptides, N-formylmethionyl-leucyl-phenylalanine (fMLP), and IL-8 (180, 181). The results of these initial studies have also demonstrated the ability of LMVs to induce IL-6, MCP-1, and TF synthesis in endothelial cells (180, 181), whereas evidence from *in vitro* studies suggests that monocyte MVs may activate other cells than endothelial cells. Furthermore, the role of monocyte-derived MVs in inflammatory response was discussed by Cerri et al. (182), who found that monocyte/macrophage MVs up-regulate secretion of inflammatory mediators, including IL-8, MCP-1, and ICAM-1, by airway epithelial cells (182). Human lung epithelial cells were subsequently reported to increase synthesis of IL-8 and MCP-1 after stimulation by monocyte/macrophage derived MVs (183). The observed effects were mediated by NF-κB activation through a peroxisome proliferator-activated receptor gamma (PPAR-γ) dependent pathway (183). Moreover, study performed by Eyre et al. (184) indicates that podocyte stimulation by monocyte MVs induces production of MCP-1 and IL-6. In this context it is particularly important that MVs released by monocytes activate the production of TNF-α and IL-6 by monocytes and macrophages (185), hence monocyte MVs work in an autocrine and paracrine mode, like EMVs. Also, monocyte and T cell MVs might be an important element in the regulation of cyclooxygenase 2 (COX-2), microsomal prostaglandin E synthase 1 (mPGES-1), and prostaglandin E_2_ (PGE_2_) production (186). In synovial fibroblasts, MVs derived from monocytes and T cells induce synthesis of inflammatory mediators (IL-6, IL-8, MCP-1, and−2) (187). To conclude, we can say that MCP-1 is the subject of many research projects concerning linking inflammation to monocyte MVs. Moreover, MVs originated from apoptotic monocytes induce ROS generation via p38 MAPK pathway in endothelial cells and enhance platelet adhesion to endothelium (188). However, in later observations, apoptotic monocyte MVs were shown to have no effect on oxidative stress (189). Given the cardinal role of ROS in inflammation (190), it is understandable that these relationships should be further examined in future studies. Furthermore, and probably more appealing to the physicians, an extensive series of clinical studies was conducted to evaluate the significance of monocyte MVs in pathogenesis of inflammation-associated disorders. It was confirmed that they constitute important elements in pathogenesis of acute myocardial infarction (92, 191), type 2 diabetes mellitus and its complications (192–194), rheumatoid arthritis (195), intracerebral hemorrhage (196), and lung cancer (197). Possible role of LMVs in sepsis has also been suggested by the finding that their levels were increased significantly in patients with severe infection (198). Some data additionally suggest that LPS stimulates the release of MVs from monocytic cell lines (199), which show proinflammatory properties, since mRNas of several cytokines were found to be up-regulated after LPS stimulation (199).

Neutrophil MVs are another essential constituent of inflammatory reactions. Stimulation of neutrophils results in releasing heterogenous MV populations, which contain hundreds of proteins, such as leukotriene A_4_ hydrolase (LKHA_4_), which also have proinflammatory effect (200). The authors showed that neutrophil MVs can move in response to a chemotactic gradient (200). There are several proinflammatory proteins recognized on the surface of neutrophil MV. In 2008 Pluskota et al. (201) identified Mac-1 integrin on MVs derived from stimulated neutrophils that activate platelets. In addition to platelets, the primary effect of neutrophil MVs includes interactions with endothelial cells, which undergo a number of molecular and biochemical changes (66, 200, 202, 203). An important factor to acknowledge while considering these interactions is the observation that neutrophil MVs deliver myeloperoxidase (MPO) to endothelial cells causing their injury (66, 203). Furthermore, one factor that was considered to be a potential link between neutrophil MVs and inflammation is the deposition of MVs by neutrophils on intestinal epithelial cells (IECs), which promotes epithelial injury (202). The next argument in favor of the existence of link between inflammation and neutrophil MVs comes from growing clinical and experimental evidence indicating that these MVs are generated during sepsis (204–206). For example, one *in vitro* study showed that THP-1, a human monocytic cell line, was activated after phagocytosis of neutrophil MVs isolated from patients with sepsis (205). Accordingly, clinical observations demonstrated that patients with *Staphylococcus aureus* bacteremia had higher levels of neutrophil MVs in their blood than healthy controls (204). Moreover, Prakash et al. (205) found elevated levels of neutrophil MVs in abdominal fluid from patients with sepsis and peritonitis. Despite these mentioned results, the complete mechanism of neutrophil MV action in sepsis remains unknown. In fact, Timár et al. (204) extended previous observations, showing the antibacterial effects of neutrophil MVs by inhibiting bacterial growth.

The biological function of neutrophil MVs is not limited to their role as proinflammatory agents. Therefore, the anti-inflammatory trend was independently confirmed in a number of studies. Experiments designed by Hyun et al. (207) and Lim et al. (208) demonstrated that neutrophil, monocytes and T cells deposit CD18^+^ MVs at the subendothelium during extravasation, playing a protective role by preventing vascular leakage. It is important to notice that the investigation of immunosuppressive functions of neutrophil MV set the foundations for discovering that they do not induce the release of IL-8 and TNF-α by macrophages (209). In the original paper describing these results, Gasser and Schifferli (209) clearly demonstrated an increased release of the anti-inflammatory mediator transforming TGF-β1 by neutrophil MV-stimulated macrophages. Accordingly, annexin A1 (AnxA1), which is present in neutrophil MVs, induces decrease in interaction between MVs and endothelial cells (210). Lastly, neutrophil MVs are also engaged in inflammation through their involvement in cytokine production by natural killer (NK) cells (211). By measuring the levels of pro- and anti-inflammatory proteins, Pliyev et al. (211) were able to demonstrate that neutrophil MVs reduced the release of IFNγ and TNF-α, but enhanced the release of TGF-β1. Altogether, this data suggests that neutrophil MVs might have different, also opposing functional roles in inflammatory response.

Three lines of direct evidence suggested that lymphocyte MVs play important role in promoting and inhibiting inflammatory reaction. First, current data indicates that activated T-cells generate MVs able to collaborate with many cell types. In short, it was shown that T-cell generated MVs induce synthesis of proinflammatory (TNF, IL-1β) as well as anti-inflammatory (secretory interleukin-1 receptor antagonist, sIL-1Ra) cytokines in monocytes (212). One provocative finding is that TNF and IL-1β production, unlike sIL-1Ra, is inhibited by high-density lipoproteins (HDL) (212). More recently, Carpintero et al. (213) reached an analogous conclusion by demonstrating that HDL inhibit T-cell MV-induced proinflammatory protein secretion by monocytes. Follow-up experiments (214–216) were designed to test the ability of T-cell generated MVs to activate mast cells (MCs). The first study that addresses this question indeed shows that T-cell generated MVs can initiate degranulation and cytokine (IL-8, oncostatin M) release from MCs (214). Attention should also be drawn to the new observation that T-cell generated MVs provoke MCs to produce IL-24 (215). Moreover, it appears that MC activation depends on miR-4443 provided by T-cell generated MVs (216). The main consequence of miR-4443 internalization into MCs is therefore to downregulate the protein tyrosine phosphatase receptor type J (*PTPRJ*) gene expression, leading to increased extracellular signal-regulated kinase (ERK) phosphorylation, and heightened release of IL-8 (216). Moreover, there is substantial evidence that T-cell generated MVs are involved in endothelial dysfunction, which was documented by Martin et al. (217) and Mostefai et al. (218). These authors postulated that T-cell generated MVs decrease NO production at the same time increasing ROS production in endothelial cells (217, 218). Based on the fact that T-cell generated MVs may interact with different cells, Qiu et al. (219) recognized them as the first to move in the proinflammatory cytokine release by bronchial epithelial cells (BECs). Other than describing MVs originating from T lymphocytes in terms of their direct inflammatory action, studies by Qui's team also proved that these MVs promote apoptosis of normal cells (BECs) (219, 220) and cancer cells (retinoblastoma cells) (221). Second, increased amounts of circulating T-cell generated MVs were found in patients with active chronic hepatitis C (222, 223). High levels of these MVs in blood were associated with disease severity (223) as most likely resulting from excessive fibrolytic activity of hepatic stellate cells (HSCs) after their fusion with MVs (222). Third, non-infectious inflammatory diseases also elevate blood lymphocyte MV levels. A striking increase in the levels of T/B lymphocyte MVs was apparent in polymyositis/dermatomyositis (67), systemic lupus erythematosus (224), rheumatoid arthritis (225) and non-alcoholic fatty liver disease (223). It is interesting that levels of B-cell derived MVs were significantly lower in multiple sclerosis patients than in healthy controls, although clinical importance of this phenomenon is unknown (226).

All the findings add to the conclusion that MVs released from leukocytes show multidirectional actions during the response of immune system. Their primary function appears to consist in the activation of proinflammatory response in other cell types. Their role in inflammation is also emphasized by studies showing their increased generation in infectious and inflammatory diseases.

## Exosomes in Inflammation—a Brief Presentation

The major goal of this paper is to explain the relationship between MVs and inflammation. However, it would certainly be interesting to know whether inflammatory reactions are affected by exosomes. Researches on this issue usually focus on the specific exosomal cargo and generally confirm proinflammatory and anti-inflammatory role of exosomes. First, exosomes enhance local and systemic inflammation due to the fact that they are sources of proinflammatory cytokines themselves and may also stimulate their production in different cells. These cytokines include proteins with recognized potent proinflammatory properties: TNFα, IL-6, IL-1β, IL-8, CXCL1 (cytokine-induced neutrophil chemoattractant 1, CINC-1), CCL2, PGE_2_, and enzymes for leukotriene synthesis (227–232). Second, exosomes may induce migration of granulocyte into inflamed tissues (232, 233) and promote inflammatory pathways in subsequent cells (20, 234). Third, *in vitro* and *in vivo* experiments demonstrated that exosomes induce B and T cells activation and proliferation (235–238).

Notably, currently available data strongly indicates that miRNAs associated with exosomes contributes to controlling inflammatory processes. A wide variety of miRNAs in exosomes was identified, however, they may have opposing roles in regulating inflammation (239): enhancing (240, 241) or suppressing inflammatory reactions (242–244). On this point, it is worth mentioning that exosomes released by cancer cells also have dual nature. Practically, these exosomes can promote and inhibit immune responses during cancer development and progression (245). One of the more intriguing aspects are also observations that systemic administration of exosomes suppress inflammation in animal models of diseases (246–249) and it is promising to use exosomes in regenerative medicine (250). Consequently, they may be a strategy in the treatment of inflammatory-associated disorder.

## Conclusion

**Figure 2:**
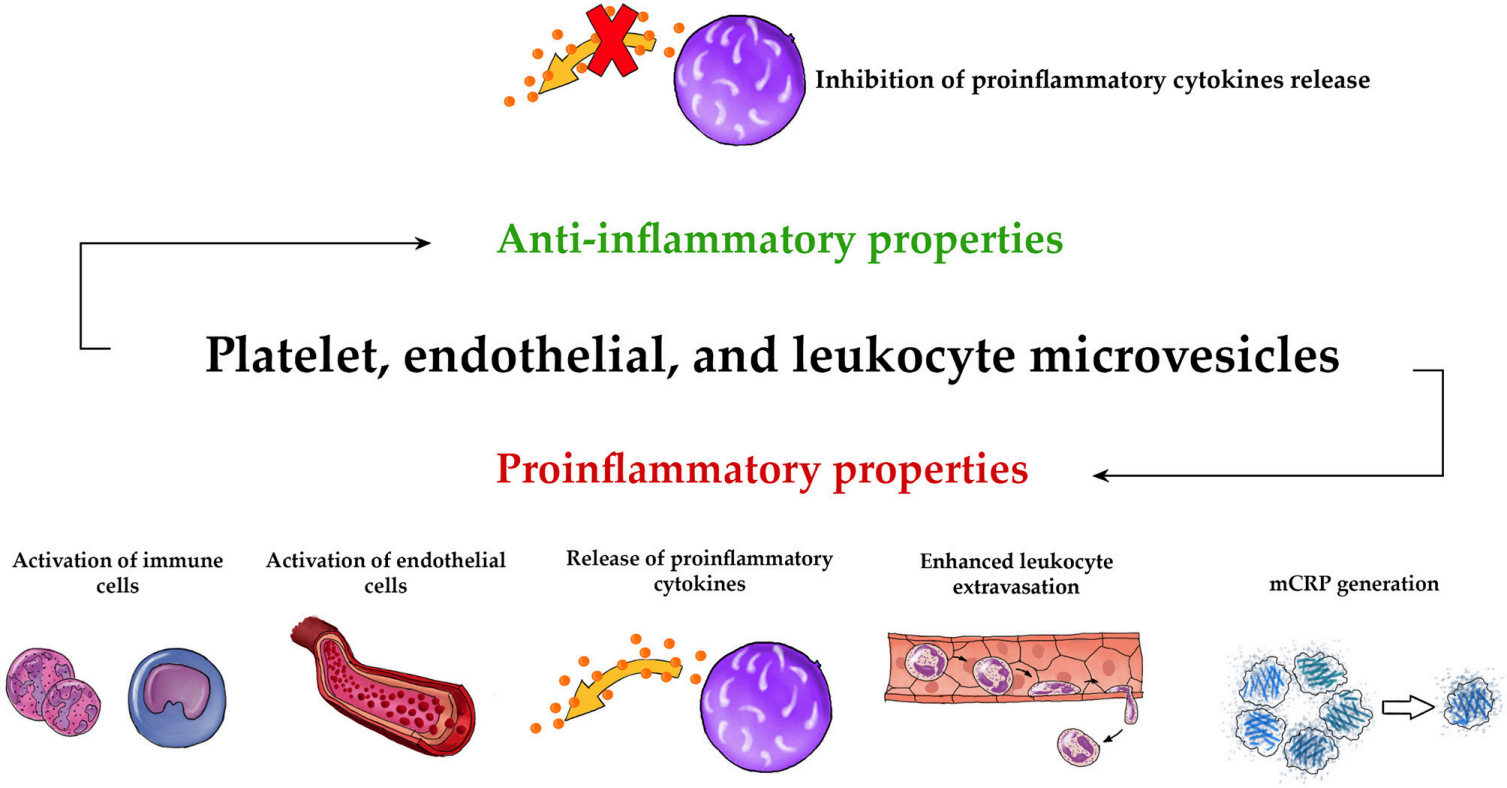
Universal pro- and anti-inflammatory properties of MVs. The three main types of circulating MVs (PMVs, EMVs, and LMVs) exhibit common proinflammatory activities such as activation of immune cells (73, 101, 105, 109, 117–119, 147, 150, 154, 156, 159, 160, 185), activation of endothelial cells (66, 72, 100, 101, 104, 108, 145, 146, 180, 181, 200, 202, 203), release of proinflammatory cytokines (111, 113, 114, 119, 121, 152–154, 180–187, 200, 201, 212, 214), enhanced leukocyte extravasation (101, 104, 108, 130, 150, 151), and mCRP generation (162, 163). They also have an anti-inflammatory effect, based on the inhibition of the release of proinflammatory cytokines (132–134, 155, 209, 210).

**Figure 3:**
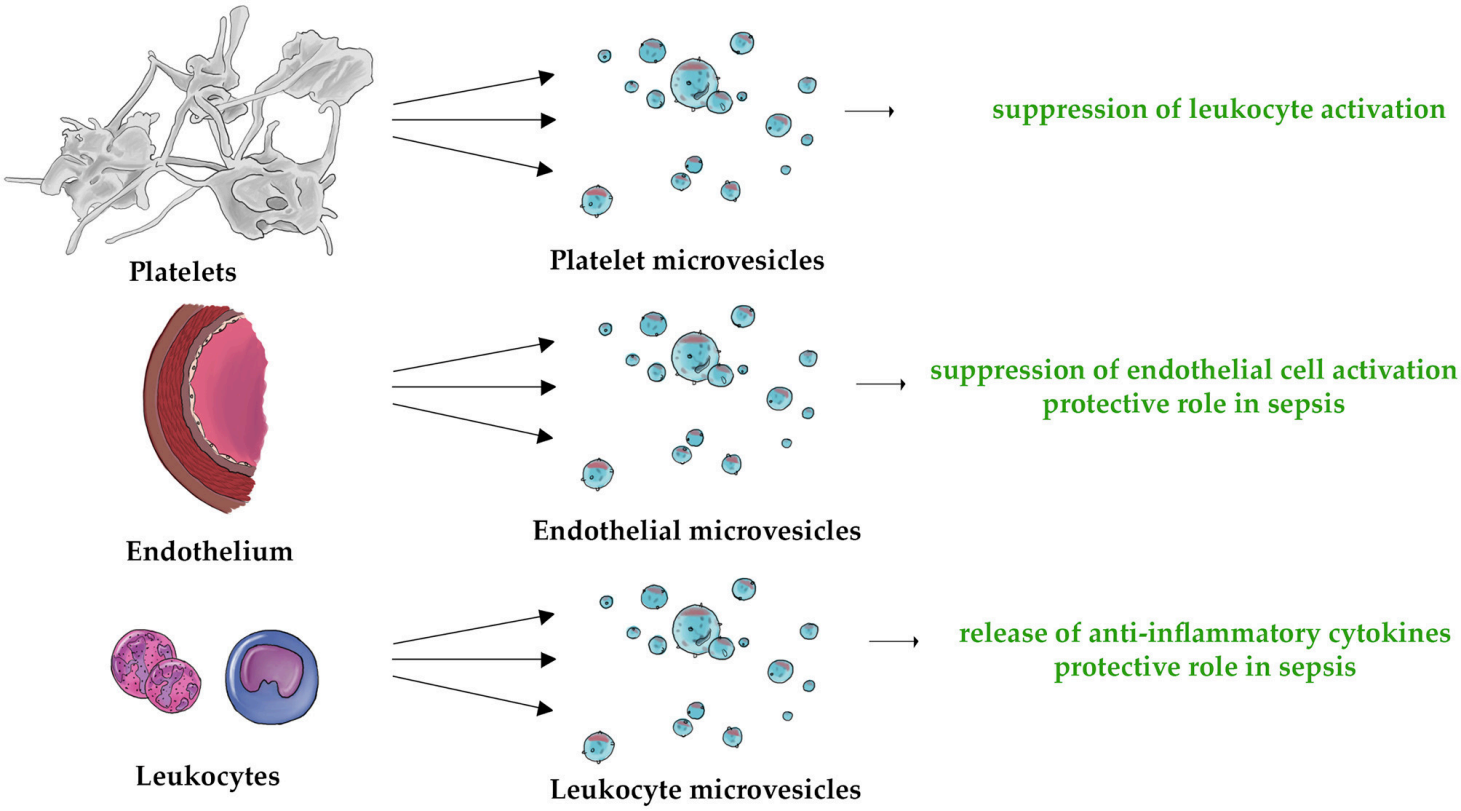
The three types of MVs (PMVs, EMVs, and LMVs) are characterized by their unique anti-inflammatory properties. This applies to the following mechanisms: suppression of leukocyte activation (132, 135), suppression of endothelial cell activation (155), protective role in sepsis (170, 171, 204), and release of anti-inflammatory cytokines (209, 210, 212).

Overall, our review strongly suggests that MVs may function as strong regulator of both innate and adaptive immune systems. Figure 2 demonstrates schematically the universal pro- and anti-inflammatory properties of PMVs, EMVs, and LMVs. The unique anti-inflammatory properties of these MVs are also shown in Figure 3. Undoubtedly, elucidation of MV functions contributes to better understanding of the complexity of inflammatory response. While studies discussed in this paper describe the importance of MVs in immunity, they also leave some significant questions unanswered.

### MVs—A New Paradox in Medicine?

Several observations indicate that MVs have paradoxical effects. They are known to coordinate significant physiological properties of tissues such as regeneration, remodeling, angiogenesis, and healing (251, 252) and may protect parental cells from lysis (253) and apoptosis (254). MVs have the ability to intensify and inhibit inflammatory processes. Concentrated researches on proinflammatory effects of MVs, especially of platelets origin, supplement current knowledge on the role of platelets in inflammation (255). Increased number of circulating MVs is a pathogenetic feature of many inflammatory diseases, which encourages researchers to explore the mechanisms of their influence on inflammation. Another example of the MVs paradox is their participation in hemostasis. Supposedly, the role of PMVs in the generation of blood hypercoagulability is well-established (256), however PMVs also exhibit anticoagulant properties (97) and bleeding results in reducing their release (257, 258). Thus, it seems that multiplicity of functions of MVs under various physiological and pathological conditions is immense and depends on specific cargo and factor stimulating their release. Hence, it is impossible to unambiguously classify MVs as beneficial or harmful structures.

### Other MV Types in Inflammation—A Brief Presentation

The interaction between numerous MV types such as red blood cells MVs (RMVs), liver MVs (hepatic MVs, HMVs), central nervous system MVs (brain MVs, BMVs), and inflammation was demonstrated by laboratory and clinical analyses. RMV forms present in both red blood cell concentrates and circulation, which some authors (259) consider to be the earliest described among all the MVs types (260), can act in a pro- and anti-inflammatory way (261–263). The liver, an organ lacking uniform histological structure (264), is able to release MVs form hepatocytes (75), cholangiocytes (265), stellate cells (265), stem cells (266), and cancer cells (267). Fundamentally, EVs released by liver cells are strongly proinflammatory (268–271). On the contrary, it was also shown that HMVs protect hepatocytes from injury (272) and induce the regeneration of parental cells (266). Populations of MVs released by CNS cells are relatively rarely studied, nevertheless, few investigations have yielded conclusions that microglia and astrocytes derived MVs carry proinflammatory IL-1β (273, 274). Opposing conclusions reached in other papers accentuate that brain MVs activate protective mechanisms in multiple sclerosis (275) and stroke (276).

### Diagnostic Potential of MVs

One critical and yet unresolved problem is the diagnostic potential of MVs in inflammatory disorders. MVs as marker for diagnosis or treatment monitoring was tested by many authors, especially in cardiovascular disorders (277) and cancer (278). Currently, many researchers define MVs, and also exosomes, as a “liquid biopsy,” which means that they can be an alternative to a classic biopsy, characterized by various limitations (278). On the other hand, however, until a precise, fast and cheap analytical method is developed, the use of MVs as a biomarker will remain fairly uncommon. One of the most widely used analytical methods for quantifying MVs levels and markers is flow cytometry (279). There are many different analytical methods used in MVs studies, such as electron microscopy (280), nanoparticle tracking analysis (280), western blot (280), dynamic light scattering (281), and enzyme-linked immunosorbent assay (282), however none of them is currently used in routine diagnostics. The vast majority of methods require special preparation of biological material samples and specialized equipment (283). In addition, recently developed sensitive and specific methods have not been yet established in the routine diagnosis yet (284). Certainly, there is an urging necessity to develop a technique that can be used in everyday clinical practice, which remains a high priority in the scope of medical care.

### MVs as Drug Delivery System

With regard to the potential use of MVs as therapeutic agents, it is realistic to expect that MVs will be exploited as a pharmacological option themselves or will prevent the development of diseases complications. Moreover, they can also be a platform for drug transport. Multidirectional actions of specific MVs cargo make that researchers deliberate over how specific therapy affects the release of MVs. Nevertheless, more evidence is obtained to confirm that predominantly exosomes perform cardinal role of therapeutic tolls, particularly in the context of anti-inflammatory (285, 286), and anticancer activity (287, 288), and are also beneficial in the treatment of CNS disorders (289, 290). Moreover, the results of clinical trials showed exosomes to be useful in treating cancers (291–293). Out of all the links between MVs and their clinical use, the interaction between MVs and cancer cells is the best documented one (294, 295). For example, a study by Tang et al. (294) showed that tumor cells incubated with chemotherapeutic drugs are likely to secrete MVs connected with drugs which are able to kill other tumor cells, but without side typical effects occurring when drugs are used alone. MVs can likewise deliver suicide mRNA/protein to cancer cells leading to tumor regression (295). Undeniably, more research evaluating such properties is necessary to provide evidence-based tools for cancer treatment.

## Author Contributions

AS performed literature search, wrote the manuscript, prepared tables, and designed figures. VL-K and EŻ supervised and critically read the manuscript. SU and MK developed the concept of the manuscript, supervised, and critically read the manuscript and designed figures.

### Conflict of Interest Statement

The authors declare that the research was conducted in the absence of any commercial or financial relationships that could be construed as a potential conflict of interest.
